# Quantitative Measurement of Melittin in Asian Honeybee Venom Using a New Method Including UPLC-QqTOF-MS

**DOI:** 10.3390/toxins12070437

**Published:** 2020-07-04

**Authors:** Sheng Huang, Jianhua Wang, Zeqin Guo, Yan Wang, Chundong Liu

**Affiliations:** Key Laboratory of Biorheological Science and Technology, Ministry of Education College of Bioengineering, Chongqing University, Chongqing 400044, China; stevenhouse@cqu.edu.cn (S.H.); guozq@cqu.edu.cn (Z.G.); wangyan1992@cqu.edu.cn (Y.W.); liuchundong@cqu.edu.cn (C.L.)

**Keywords:** Asian honeybee venom, melittin, quantitative measurement, ultra-performance liquid chromatography-quadrupole time-of-flight mass spectrometry (UPLC-QqTOF-MS)

## Abstract

Asian honeybee venom is widely used in traditional oriental medicine. Melittin is the main component of Asian honeybee venom. In the present study, an ultra-performance liquid chromatography-quadrupole time-of-flight mass spectrometry (UPLC-QqTOF-MS) method was used for accurate qualitative and quantitative analyses of melittin in Asian honeybee venom. The results showed that the dynamic linear range of melittin was from 0.094 to 20 μg/mL, and the limit of quantification was 0.3125 μg/mL. The spiking recovery of melittin in honeybee venom ranged from 84.88% to 93.05%. Eighteen Asian honeybee venom samples in eighteen batches were collected from two different zones of China, and their melittin contents were measured. The contents of melittin in Asian honeybee venom samples was 33.9–46.23% of dry weight. This method proved a useful tool for the rapid evaluation of the authenticity and quality of Asian honeybee venom in terms of the melittin contents, and will contribute to a broader understanding of Asian honeybee venom.

## 1. Introduction

*Apis cerana Fabricius* is a type of Asian honeybee whose venom has been widely applied as a traditional oriental medicine to treat some diseases such as chronic pain diseases [1] and inflammatory diseases [2]. The latest research has shown that bee venom also exhibits potential therapeutic effects toward neurological diseases, such as amyotrophic lateral sclerosis (ALS) [3] and Parkinson’s Disease (PD) [4,5], in addition to peripheral neuropathy [6,7,8], cancer [9,10,11], and circulatory diseases [12]. Bee venom is a complex mixture consisting of enzymes such as phospholipase A2 (PLA) and hyaluronidase, peptides such as melittin, apamin, and mast cell degranulating peptide, inorganic salts, lipids, amino acids, and other compounds [13].

Melittin is the main component of bee venom and exhibits many excellent biological activities such as antibacterial [14,15,16], antiviral [17,18], and anticancer properties [19,20,21]. Researchers have also detected melittin in the body surface of bees and comb wax [22], and speculated that it might be involved in the community immunity of the honeybee [23], thereby indicating that melittin also plays an essential role in the biology of bees. Several previous studies have focused on analyzing the content of melittin in Western honeybee (*Apis mellifera*) venom, and it has been found that the content of melittin can exceed 50% of the dry weight of bee venom [24,25,26,27,28]. However, few reports exist into the accurate detection of content of melittin in Chinese honeybee (*Apis cerana Fabricius*) venom. The development of a convenient analytical method with high selectivity and sensitivity is, therefore, necessary for this purpose, and could be useful in investigating the role of melittin in Chinese bee biology, in addition to evaluating the authenticity and quality of Chinese honeybee venom products.

Several analytical methods have been previously developed for the quantitative measurement of melittin in bee venom from the Western honeybee (*Apis mellifera*). Among those methods, reversed-phase high-performance liquid chromatography (RP-HPLC) has been commonly employed to separate melittin, and this has been followed by detection using diode array detector (DAD) [25], ultraviolet (UV) detector [24], photodiode array detector (PDA) [28], and tandem mass spectrometry (MS/MS) [29,30]. Compared to MS, the DAD, UV, and PDA detectors have limited capacities for the characterization of melittin in bee venom because of their lower selectivities and sensitivities. However, the selectivity of the tandem quadrupole mass spectrometers used in previous methods was not sufficient to accurately characterize the identity of melittin in bee venom due to the low mass resolution, resulting in a higher limit of quantification (LOQ; 1 μg/mL) [29]. However, by lowering the LOQ of the detection method, accurate detection of the melittin content in bee venom products should be possible. Compared to quadrupole MS, techniques based on high-resolution mass spectrometry (HRMS), such as quadrupole time-of-flight mass spectrometry (QqTOF-MS), allow accurate mass assignments by deriving a narrow mass extraction window to resolve mass isobaric species from complex mixtures and enable precise identification. These instruments can perform a ’parent-to-parent’ method for quantification to eliminate the reduced fragmentation [31]. These advantages result in improved selectivities and sensitivities in the context of bioanalytical analysis, especially in the case of biomacromolecules [32]. Modern HRMS instruments can also perform quantitative analysis in the multiple reaction monitoring (MRM)-like mode, which has been widely used for the simultaneous characterization, comprehensive qualitative exploration [33,34], and quantitation of peptides and proteins [35,36].

Thus, in an attempt to better understand the content of melittin in Chinese honeybee venom, we herein report the development of a new method to simultaneously characterize and quantify melittin in Chinese honeybee venom using an ultra-performance liquid chromatography-quadrupole time-of-flight mass spectrometry system (UPLC-QqTOF-MS). The developed method should not only determine the accurate mass for confident identification of melittin but should also allow the quantitation of melittin via the MRM^HR^ (high-resolution) mode. To the best of our knowledge, this is the first report in the analysis of melittin in Chinese honeybee venom through UPLC-QqTOF-MS.

## 2. Results

### 2.1. Identification of the Precursor and Fragment Ions of Melittin

**Figure 1:**
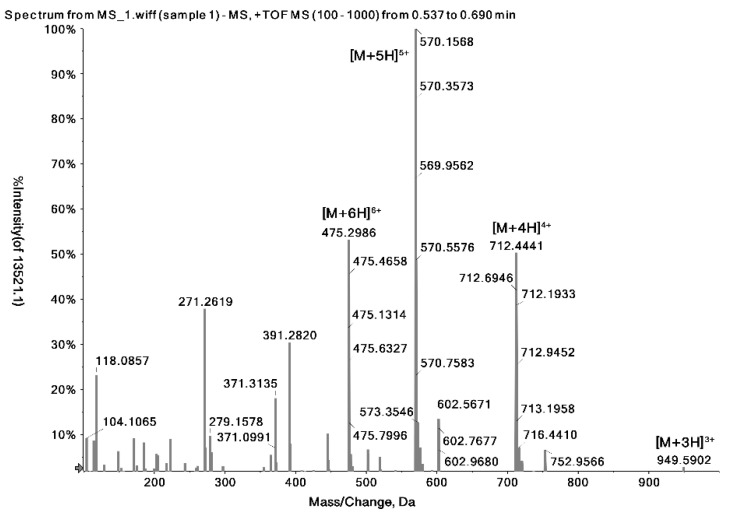
High-resolution full scan mass spectrum of melittin.

Melittin is a polar peptide that can be protonated at low pH values for positive ion mode detection by MS [37]. Figure 1 shows a typical high-resolution mass spectrum of melittin in the MS full scan mode with a narrow extract window (±10 mDa). The ions at m/z 949.5902, 712.4441, 570.1571, and 475.2986 can be assigned to [M + 3H]^3+^, [M + 4H] ^4+^, [M + 5H]^5+^, and [M + 6H] ^6+^, respectively (Table 1). These high-resolution multiply charged ions can be used to identify the melittin present in the bee venom sample more accurately.

The ion at m/z 570.1571 ([M + 5H]^5+^) was found to be the most abundant, and so was selected as the precursor ion to conduct a product ion scan. The second most abundant ion at m/z 712.4441 ([M + 4H]^4+^) was used as the confirmation ion of melittin in MRM quantitative analysis. 

**Figure 2:**
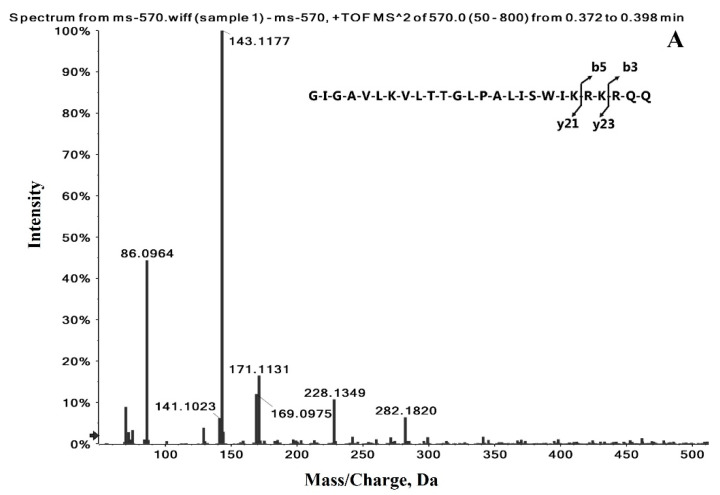

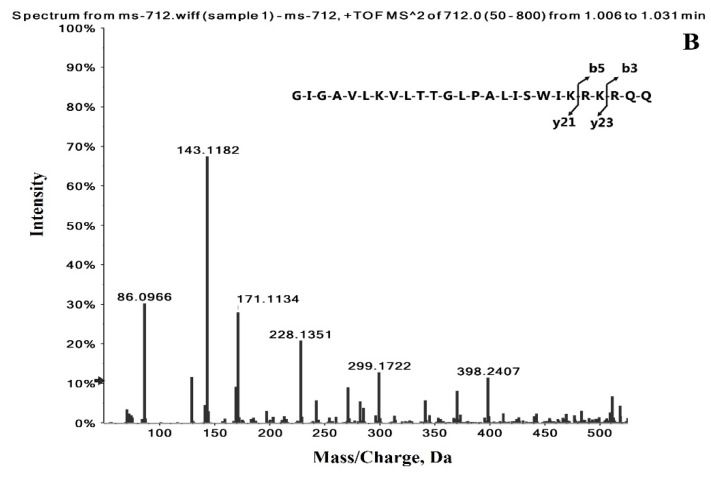
Fragment ion spectra formed from precursor ions with m/z (**A**) 570 and (**B**) 712.

Figure 2 shows the spectra of product ion scans for the ions at m/z 570.1571 ([M + 5H]^5+^) and m/z 712.4441 ([M + 4H]^4+^). The fragment ions at m/z 86.0964 and m/z 143.1177 were found to be the most abundant (Figure 2A), where the ion at m/z 86.0964 can be assigned to a loss of y23 (GIGAVLKVTTGLPALISWIKRK) amino acid residues from the protonated melittin ion, while that at m/z 143.1177 can be assigned to a loss of y21 (GIGAVLKVLTTGLPALISWIK) amino acid residues from the C-terminal side. The two ions at m/z 86.0964 and m/z 143.1177 were also observed in the product ion spectrum of the ion at m/z 712 (Figure 2B), which can be used to confirm the identity of melittin in MRM quantitative analysis. The transition m/z 570.1571→143.1177 was used for quantitative analysis, and the transition m/z 712.4441→143.1177 was used for confirmation. These high-resolution product ions not only increased the selectivity of the quantitative process but also limited the effects of high chemical noise and isobaric interference, ultimately improving the overall sensitivity of the quantitative analytical technique. 

### 2.2. Chromatographic Profiles of Melittin 

**Figure 3:**
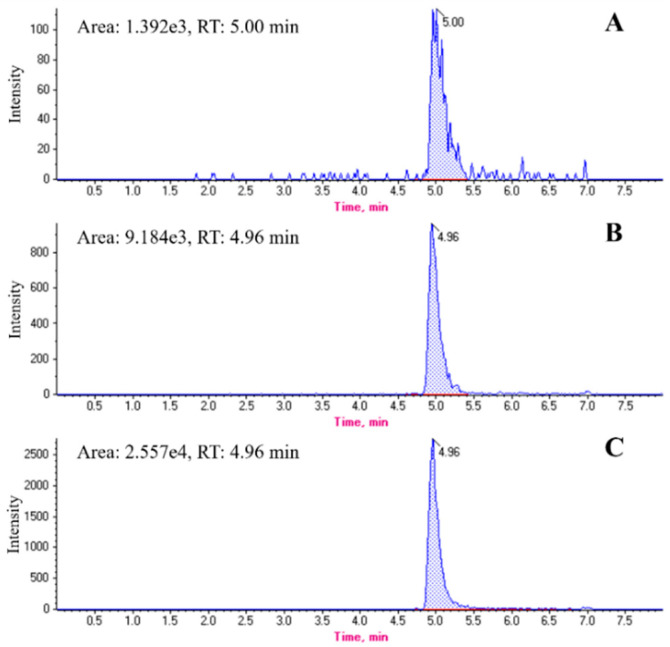
Representative MRM-like chromatograms: (**A**) 0.3125 μg/mL of melittin solution; (**B**) quality control (QC) samples spiked with 0.3125 μg/mL of melittin; and (**C**) real bee venom sample.

Based on the above experimental results, the typical MRM-like (m/z 570.1571→143.1177) chromatograms for melittin and real bee venom generated on the instrument are shown in Figure 3. 

### 2.3. Method Validation

#### 2.3.1. Linearity and the Matrix Effect

In this study, two series of calibration solutions, i.e., solvent-based (set 1) and matrix-based (set 2), were prepared to obtain two calibration curves. Excellent linearities (R_2_ > 0.99) were obtained for both the calibration curves (Table 2).

#### 2.3.2. Limit of Detection (LOD) and LOQ

The LOD of this method was determined to be three times the signal to noise ratio (S/N = 3) of the lowest concentration of melittin in real bee venom, and the LOQ was established to be ten times the signal to noise ratio (S/N = 10). According to these definitions, the LOD and LOQ of the method were 0.094 and 0.3125 μg/mL, respectively.

#### 2.3.3. Accuracy and Precision

The method precision was evaluated using the relative standard deviation (RSD) of replicate measurements which was found to be in the ranges of 4.6–9.5% for intra-day and 4.7–10.6% for inter-day (Table 3). These results indicated that the accuracy and precision of this method were satisfactory. 

#### 2.3.4. Melittin Content in Asian Honeybee Venom

The developed method was then applied to measure the melittin contents in bee venom samples collected from Wuhan and Jilin, with nine batches being obtained from each location. The mean value of melittin in each batch (total 18 batches) was 33.9–46.2% of dry weight in the bee venom of *Apis cerana* from two areas of China (Table 4).

## 3. Discussion

Traditionally, ligand-binding assays (LBAs) and triple quadrupole-based assays are employed for the quantitative detection of large molecules [38]. However, the above two methods have their shortcomings in the quantification of macromolecules, which can be solved by liquid chromatography (LC)-HRMS. For example, in the case of LBAs, the HRMS instrument can address disadvantages such as time-consuming method development and cross-impact issues. Besides, in the context of triple quadrupole-based assays, HRMS can overcome the limitations in the quantification of biomacromolecules because it offers higher resolution, higher acquisition speed, and broader mass range. Furthermore, with the launch of new time-of-flight (TOF) components, these detectors can simultaneously and accurately identify the molecules present in the matrix and sum the multiple product ions for quantitative determination. For example, due to the fact that it offers an excellent paradigm of sensitivity and selectivity, the high-resolution MS-like QqTOF-MS has begun to revolutionize the quantitative bioanalysis industry. Nowadays, the quantitation of large molecules such as peptides, proteins, and polysaccharides using HRMS is becoming more prevalent, as verified by multiple publications [39,40,41].

In the context of our study, we considered it necessary to adjust the UPLC conditions for analyzing the melittin in bee venom, especially in terms of the choice of additives for the mobile phase, because additives can aid in increasing separation [42], in addition to improving the response of high-level buffers. Common mobile phase additives include formic acid, trifluoroacetic acid (TFA), ammonium formate, and ammonium hydroxide, where formic acid and TFA are commonly used to enhance the ionization efficiency of peptides and proteins in the LC separation [43,44,45]. However, we found that TFA significantly suppressed the melittin signal, likely due to the effect of ion-pairing, which suppresses the ionization of melittin [46,47,48]. In contrast, no such signal suppression was observed for formic acid, and a better peak shape was obtained, in addition to shorter run time. Therefore, 0.1% of formic acid was used as the solvent modifier in the mobile phase. Figure 3 shows the melittin peaks of the bee venom chromatograms obtained both with and without the spiking of melittin under the optimized conditions.

Low-resolution MS-like triple quadrupole instruments are commonly considered the gold standard for quantitative analysis. However, in some cases, low-resolution nominal MS instruments are not suitable due to high background or noise matrix interference, which can result in a high LOQ and a high coefficient of variation (CV). In an earlier study [29], an HPLC-DAD-tandem MS method was used to determine the content of melittin in bee venom from *Apis mellifera*. The results showed that the LOQ of this method was 1.0 μg/mL, and the precision results (expressed as a CV) were as high as 11.4% for the intraday repeatability and 13.1% for the interday intermediary precision. In our study, we initially employed the QqTOF-MS system in the full scan mode to acquire the exact mass of melittin, after which the system was switched to MRM^HR^ (high-resolution) mode to collect the quantitative information for melittin. This scan mode was used to quantify the fragment ion of melittin with a high resolution to reduce background noise. By applying these two scanning modes, the established method significantly reduced the LOQ to 0.3125 μg/mL. Moreover, the method precision ranged from 4.6% to 9.5% for intra-day precision, and from 4.7% to 10.6% for inter-day precision. These experimental results indicate an improved precision compared to that by low-resolution MS system.

Ion suppression in electrospray ionization (ESI) source-based HPLC-MS/MS systems is an ongoing challenge in the quantitative analysis of biological samples. This phenomenon may be caused by the interference of the co-eluting compounds [49], or the mobile phase additives [50], and can affect the reliability of the final analytical results. Therefore, analysts must adequately evaluate this, and either avoid or reduce its impact. The most common means to assess the ion suppression of any matrix in a sample is to compare the slopes of two calibration curves, which are obtained using the mobile phase and the matrix. If ion suppression is present, this can be observed by differences in the slopes of these two curves. Once ion suppression is observed in a sample matrix, the most crucial step is to reduce or remove the interference. To date, several methods have been reported to remove or reduce matrix effects, including the development of a selective extraction method [51,52], adjusting the chromatographic method to completely separate the co-eluated components from the analytes of interest [53], and diluting the sample to reduce the matrix effects [54,55]. In this study, a particularly facile and straightforward sample dilution method was selected to reduce the matrix effect in the quantitative analysis of melittin in the bee venom. More specifically, the prepared bee venom extract was subjected to 100-fold dilution with a 0.1% formic acid solution for sample analysis by a UPLC-QqTOF-MS system, and two calibration solutions, i.e., solvent-based (series 1) and matrix-based (series 2) solutions, were prepared to obtain two calibration curves. The intercept of calibration curve 2 was higher than that of calibration curve 1, thereby indicating the presence of endogenous melittin in the pooled bee venom samples. The slopes of both calibration curves (1 and 2) were similar (2880.54 ± 2.3 vs. 2845.86 ± 1.8), indicating that the matrix effect from the bee venom matrices was minimal when this sample dilution method was employed. The dynamic linear range of melittin was found to be 0.094–20 μg/mL, and so this large linearity for the HRMS process indicated its applicability for the analysis of various melittin-containing bee venom samples.

From our results, it is apparent that the content of melittin in the bee venom samples obtained from the two different regions (Wuhan and Jilin) varies throughout the year. More specifically, the highest melittin content in the Wuhan samples was detected in June, while the highest melittin content in the Jilin samples was found in August. Bee venom should be collected in mid-June in the Wuhan area, while in the Jilin area, the collection should be postponed until August to obtain higher quality bee venom products. We, therefore, speculate that the melittin content in bee venom was influenced by seasonal variations. In another report, Junior et al. [34] studied the changes in the content of melittin in bee venom (from *Apis mellifera*) with the season, temperature, humidity, rainfall, and hours of sunlight, in addition to other factors. Their results suggested that the content of melittin indeed correlated with the seasonal changes, which agreed with our results. However, the melittin content in our bee venom samples was found to be slightly lower than that reported by other groups, such as the Sarker group [33], who detected a melittin content of 59.3% (c.f. 46.23% as our highest value). We hypothesized that the difference in results could potentially be due to differences in collection times, sampling locations, or other factors. However, previous research into the melittin contents in *Apis mellifera* venom suggests that a comparison of the production of melittin in *Apis cerana* and *Apis mellifera* from the same geographical location (i.e., Wuhan and Jilin) should be made over a year to determine the source of this variation.

## 4. Conclusions

We herein described the development of a method for the quantitative measurement of melittin in Asian honeybee venom (*Apis cerana*) using ultra-performance liquid chromatography-quadrupole time-of-flight mass spectrometry (UPLC-QqTOF-MS). This new UPLC-QqTOF-MS methodology allowed the accurate identification and quantitation of melittin in the bee venom samples. Additionally, it was found that the sensitivity of the method increased significantly with an increase in selectivity. Furthermore, a broad dynamic linear range for melittin detection was obtained, i.e., from 0.094–20 μg/mL, thereby indicating the applicability of this method in the analysis of different melittin-containing bee venom samples, whereby the melittin content can be used as a quality indicator for medical products. This method could also lead to advances in the evaluation of the biology of *Apis cerana*. Overall, our study constitutes the first report providing specific and accurate data to expand our understanding of the content of melittin in Asian honeybee venom. 

## 5. Materials and Methods 

### 5.1. Chemicals and Reagents

Melittin (amino acid sequence: GIGAVLKVLTTGLPALISWIKRKRQQ-NH2, MW = 2846 Da) was purchased from Sigma–Aldrich (Sigma–Aldrich Shanghai Trading Co Ltd, Shanghai, China). Acetonitrile and methanol were obtained from Merck (Merck KGaA, Darmstadt, Germany, LC-MS grade). Deionized water (DI; 18.25MΩ·cm) was obtained in-house using a Direct-Q 3 Millipore Ultrapure water purification system (Millipore, Burlington, MA, USA). Formic acid (Merck KGaA, Darmstadt, Germany; analytical reagent grade) was used as the pH modifier.

### 5.2. Preparation of the Working and Calibration Standard Solutions

The melittin stock solution (1000 μg/mL) was prepared by dissolving the necessary quantity of standard melittin powder in a 0.1% aqueous formic acid solution and was stored at 4 °C before use. The working solution (200 μg/mL) was prepared by diluting the melittin stock solution with the 0.1% aqueous formic acid solution and storing at 4 °C before using. 

The standard calibration solutions with concentrations of 0.3125, 0.625, 1.25, 2.5, 5, 10, and 20 μg/mL were prepared by serially diluting the working solution with the 0.1% aqueous formic acid solution. The matrix-based calibration standard solutions (0, 0.3125, 0.625, 1.25, 2.5, 5, 10, and 20 μg/mL) were prepared through the spiking of melittin working solutions in the quality control zero-level (QC0) sample. 

### 5.3. Preparation of the Quality Control (QC) Samples

The QC samples were prepared by pooling and mixing a portion of each above-collected bee venom powder sample. An aliquot (1 mg) of the pooled bee venom powder was weighed and transferred to 2 mL microcentrifuge tube. Subsequently, 0.1% aqueous formic acid solution (1 mL) was added to the microcentrifuge tube to dissolve the powder. The above solution was subjected to sonication for 15 min, followed by vortexing for 5 min. The mixture was then subjected to centrifugation at 10,000 rpm for 15 min at room temperature (25 °C), after which the supernatant was transferred to a 2 mL microcentrifuge tube. The non-spiked pooled bee venom sample (diluted 100-fold with 0.1% aqueous formic acid solution) was used as the QC0 sample. Recovery control samples at three levels, i.e., quality control low-level (QCL), quality control mid-level (QCM), and quality control high-level (QCH), were prepared by spiking the melittin standard solution into the QC0 samples. The concentrations of spiked melittin were 1, 5, and 10 μg/mL, respectively. 

The QC samples were prepared in replicate (*n* = 5), and the mean recovery, RSD of intra-day repeat measurements (*n* = 5), and RSD of inter-day repeat measurements (*n* = 15, three consecutive working days) were used to evaluate the method accuracy and precision.

### 5.4. Collection and Preparation of the Bee Venom Samples

Eighteen bee venom (from *Apis cerana*) lyophilized powder samples (18 batches) were purchased from Wuhan Yimin Bee Products Co., Ltd. and the Apiculture Science Institute of Jilin Province (Jilin, China), with nine batches being obtained from each location. The bee venom samples were collected from two different cities, namely Wuhan and Jilin, as follows. The venom collector consisted of a pulse, an electric grid, and a glass plate. The output voltage of the electric grid was set to 3 V and was paused automatically. The venom collector power was switched on to knock the hive to irritate the worker bee. When the bee touched the electric grid, the bee venom was deposited on the glass plate through the bee sting needle. Following the volatilization of the bee venom liquid to the crystalline state, the dried bee venom was scraped off from the glass plate and stored at –20 °C before using.

The bee venom powder sample was then allowed to warm to room temperature, and an aliquot (1 mg) was weighed and transferred to a 2 mL microcentrifuge tube. Subsequently, a 0.1% aqueous formic acid solution (1 mL) was added to the microcentrifuge tube to dissolve the powder. The resulting venom solution was subjected to sonication for 15 min, followed by vortexing for 5 min. The mixture was then subjected to centrifugation at 10,000 rpm for 15 min at room temperature (25 °C). After filtration of the supernatant using a 0.22 μm membrane, it was diluted 100-fold with 0.1% aqueous formic acid solution for sample analysis using the UPLC-QqTOF-MS system.

Samples were analyzed batch by batch. In each batch, there were two blanks, two QC0, two QCL, two QCM, two QCH, and six bee venom samples randomly selected from the 18 samples, three each from two locations.

### 5.5. Instrumentation and Analytical Conditions

A Nexera UPLC LC-30A system (Shimadzu, Kyoto, Japan) was coupled with a Sciex Triple TOF 4600 system (AB Sciex, Framingham, MA, USA) equipped with a Turbo V ESI source to conduct the sample analysis. Analyst^®^TF 1.6 software (AB Sciex, Framingham, MA, USA) was used to control both the UPLC and QqTOF-MS systems. The pressures of the sheath gas (zero air) and the auxiliary gas (zero air) were 50 and 10 Psi, respectively. The pressure of the curtain gas (nitrogen) was 25 Psi, the atomizing temperature was 600 °C, the full scan resolution was 30,000 FWHM at 956 m/z, and the MS/MS scan resolution was 25,000 FWHM at 195 m/z. The scan range in full scan mode was from m/z 100 to 1000, and the accumulation time for a full scan was 250 ms. Dynamic background subtraction (DBS) was used as the second trigger condition, and the information-dependent acquisition (IDA) MS/MS mode was used to collect the information relating to the melittin product ions. The accumulation time of an MS/MS scan was 100 ms. The declustering voltage and collision energy (CE) collision energy were 100 V and 35 eV, respectively. The MRM^HR^ mode was used to collect quantitative information relating to the melittin. The mass extraction windows were set as ±10 mDa. PeakView^®^version 1.2 with XIC Manager (AB Sciex, Framingham, MA, USA) was used to identify the peaks from the MS scan results, and MultiQuant™ software version 2.1 (AB Sciex, Framingham, MA, USA) was used for quantitative analysis. Instrument calibration was carried out each day before analysis to ensure the mass accuracy of the TOF detector and the laboratory where the instrument was placed was controlled at a constant temperature of 22 °C to avoid mass drift during mass calibration.

Separation was carried out using a Bio-C18 column (50 × 2.1 mm, 5 μm, 300 Å, Sepax Technologies, Newark, DE, USA). Mobile phase A consisted of 0.1% formic acid in water, while mobile phase B consisted of 0.1% formic acid in acetonitrile. The linear gradient started from isocratic 5% B for 0.2 min, then increased to 85% B over 4.3 min, holding for 1.5 min, and then switching back to 5% B over 1.9 min. The equilibrium time was 2 min. The column temperature was set at 30 °C, the flow rate was 0.35 mL/min, and the injection volume was 5 μL. A peak with a retention time within 5% of variation compared to the corresponding standard in the calibration curve was considered positive. Between sample injections, the injection needle was washed three times using a 0.1% aqueous formic acid solution to eliminate carryover.

### 5.6. Method Validation 

The developed UPLC-QqTOF-MS method was evaluated in terms of its linearity, matrix effect, LOD, LOQ, precision, and accuracy.

#### 5.6.1. Linearity and the Matrix Effect

The linear correlation coefficient (R2) should be higher than 0.99. Calibration curve 1 was obtained by plotting the peak area of melittin in a 0.1% aqueous formic acid solution against the corresponding concentrations. Calibration curve 2 was obtained by plotting the peak area against the melittin concentrations in the QC samples using a linear regression model. The matrix effect was assessed by comparing the slope derivation from calibration curves 1 and 2. 

#### 5.6.2. LOD and LOQ

The LOD of this method was defined as three times the signal to noise ratio (S/N = 3) of the lowest concentration of melittin in real bee venom, and the LOQ was defined as ten times the signal to noise ratio (S/N = 10).

#### 5.6.3. Precision and Accuracy

The mean recovery of melittin spiked in the QC samples was calculated to evaluate the method accuracy. More specifically, the accuracy was evaluated by comparing measured concentrations with spiked concentrations (RSD %), and should be <15% for triplicate QC samples. Finally, the intra- and inter-day precisions were evaluated using the RSDs of replicate measurements.

## Figures and Tables

**Table 1:** Name, molecular weight, amino acid sequence, and most abundant charge states of melittin.

Peptide Name	Molecular Weight (Da)	Sequence	Formula	Observed Charge States	Most Abundant Isotopes Mass (Da)
Melittin	2846.46	GIGAVLKVLTTGLPALISWIKRKRQQ	C131H229N39O31	+3	949.5902
				+4	712.4441
				+5	570.1568
				+6	475.2986

**Table 2:** Analytical parameters for the melittin calibration curves.

Analyte	Calibration Curve Type	Slope (a ± Sa)	Intercept (b ± Sb)	Regression Coefficient (r)
Melittin	1	2880.54 ± 2.3	16,066.18 ± 4.7	0.99977
	2	2845.86 ± 1.8	22,401.19 ± 5.7	0.99731

**Table 3:** Recovery, accuracy, intraday precision, and interday precision for melittin in the quality control (QC) samples obtained at three different concentrations.

Spiked Level (μg/mL)	Mean Recovery in Intra-Day SD (*n* = 5, %)	Intra-Day RSD (*n* = 5, %)	Mean Recovery in Inter-Day (*n* = 15, %)	Inter-Day RSD (*n* = 15, %)
1	87.35 ± 8.3	9.5	84.88 ± 8.9	10.6
5	91.65 ± 4.0	5.4	92.32 ± 5.7	6.2
10	90.14 ± 4.2	4.6	91.07 ± 4.3	4.7

Notes: SD and RSD are abbreviations of standard deviation and relative standard deviation, respectively.

**Table 4:** Melittin contents (% dry weight) in the bee venom samples of *Apis cerana* from two areas in China.

Sampling Location	Sampling Date	Batch Number	Content (%)	Daily Mean Content (%)	SD
Wuhan	28 April 2015	W-1	41.8	40.53	0.95
		W-2	39.5		
		W-3	40.29		
	14 June 2015	W-4	44.3	46.23	1.40
		W-5	47.6		
		W-6	46.79		
	14 August 2015	W-7	37.91	37.27	1.42
		W-8	38.6		
		W-9	35.3		
Jilin	19 May 2015	J-1	35.6	33.9	1.35
		J-2	32.3		
		J-3	33.8		
	24 August 2015	J-4	43.2	44.73	1.58
		J-5	46.9		
		J-6	44.29		
	9 October 2015	J-7	35.51	34.77	1.87
		J-8	36.6		
		J-9	32.2		
